# The role of breeding system in community dynamics: Growth and mortality in forests of different successional stages

**DOI:** 10.1002/ece3.4190

**Published:** 2018-06-27

**Authors:** Yunyun Wang, Robert P. Freckleton, Bojian Wang, Xu Kuang, Zuoqiang Yuan, Fei Lin, Ji Ye, Xugao Wang, Zhanqing Hao

**Affiliations:** ^1^ CAS Key Laboratory of Forest Ecology and Management Institute of Applied Ecology Chinese Academy of Sciences Shenyang China; ^2^ Department of Animal and Plant Sciences University of Sheffield Sheffield UK; ^3^ Institute of Ecology and Key Laboratory for Earth Surface Processes of the Ministry of Education College of Urban and Environmental Sciences Peking University Beijing China; ^4^ University of Chinese Academy of Sciences Beijing China; ^5^ China School of Life Sciences and Engineering Henan University of Urban Construction Pingdingshan China

**Keywords:** breeding system, demographic rate, forest succession, growth rate, mortality rate, temperate forest

## Abstract

Plant sexual systems appear to play an important role in community assembly: Dioecious species are found to tend to have a higher propensity to colonize communities in early successional stages. Here, we test two demographic hypotheses to explain this pattern in temperate forests. First, we test demographic differences between hermaphrodite and dioecious species in stressful younger successional stages: Previous theory predicts that hermaphrodite seed production is more harmed in stressful environments than that of dioecious populations leading to an advantage for females of dioecious species. Second, in primary forest, we hypothesized that dioecious species would show demographic advantage over monomorphic ones. We used data from two temperate forest plots in Northeast China surveyed over 10 years to compare the rates of growth and mortality of tree species with contrasting breeding systems in both secondary and primary forests. We assessed the effect of breeding system on the growth‐mortality trade‐off, while controlling for other traits usually considered as correlates of growth and mortality rates. We show that in the secondary forest, dioecious species showed weak advantage in demographic rates compared with monomorphic species; dioecious species showed considerably both lower relative growth and mortality rates compared to the hermaphrodites in the primary forest over 10 years, consistent with a priori predictions. Hermaphrodites showed strong growth‐mortality trade‐offs across forest stages, even when possibly confounding factors had been accounted for. These results suggest that sexual system influences community succession and assembly by acting on the rates of growth and mortality, and the trade‐off between them. As vegetation develops, the demographic differences between breeding systems are much larger. Our results demonstrate the association between breeding system, succession, and community assembly and that this relationship is succession‐stage dependent. Our findings support the suggestion that the demographic advantage of dioecious species facilitates the coexistence of sexual systems in primary forest.

## 1 INTRODUCTION

Sexual systems are key determinants of the ecological and evolutionary dynamics of plant populations (Barrett & Harder 1996; Charlesworth, 2006) and their links to the succession and assembly of plant communities have gradually become the focus of researchers recently (Queenborough et al., 2009; Sinclair, Korte, & Freeman, 2013; Réjou‐Méchain & Cheptou, 2015; Bruijning et al., 2017). In an ultimate manner, the dynamics of plant communities are driven by plant demographic rates (growth and mortality; Pacala et al., 1996; Rees, Condit, Crawley, Pacala, & Tilman, 2001; Silvertown, 2004). Differences in these rates between species are major determinants of ecological succession (Schneider, Ashton, Montagnini, & Milan, 2014) and stand structure (Harper, 1977; Seifert, 2014; Semwal, Nautiyal, Maikhuri, Rao, & Saxena, 2013). In terms of sexual system, it has been found that the dioecious species tend to have a higher propensity (proportion of dioecious species or individuals) to colonize communities in early successional stages (temperate in Sinclair et al., 2013; tropical in Réjou‐Méchain & Cheptou, 2015), however, the drivers generating this pattern remain unclear. Thus, there is a need to understand how breeding systems drive demographic rates across communities at different stages of succession.

An evolutionary‐driven association between dioecy and colonization ability has been proposed to be a potential advantage of dioecy over cosexual breeding systems in young successional areas, which are typically characterized by stressful conditions (Réjou‐Méchain & Cheptou, 2015; Sinclair et al., 2013). Young successional forest stands which have been subject to logging, fire or other disturbance, are usually drier, have higher insolation, and are hotter, because early plant communities have small stature, offer little cover, have small basal area and a small leaf area index (Alvarez‐Añorve, Quesada, Sánchez‐Azofeifa, Avila‐Cabadilla, & Gamon, 2012; Lebrija‐Trejos, Meave, Poorter, Pérez‐García, & Bongers, 2010). That is, under stressful conditions, hermaphrodite seed production may be more impeded than the female seed production of dioecious populations, leading to a demographic advantage of females over hermaphrodites (Ashman, 2006; Costich, 1995; Litrico, Pailler, & Thompson, 2005). This hypothesis has been often tested in gynodioecious populations (Ashman, 2006), but community‐level studies are lacking: If this hypothesis is correct then at the community level, we predict dioecious species should have an advantage in demographic performance (in terms of growth and survival) over monomorphic species in the earlier successional stages.

As succession proceeds, plants from late successional stages tend to be longer‐lived and more competitive (Bazzaz 1979; Prach et al. 1997; Garnier et al., 2004; Vile et al. 2006). In stable primary forest, theoretical models suggest that dioecious seedlings tend to be more competitive than cosexual ones and that this benefits the persistence of dioecy (Heilbuth et al. 2001). This is because dioecious species face relatively more intense density‐dependent recruitment of seedlings: only females produce seeds and hence seeds of dioecious species are more spatially clumped (Heilbuth, Ilves, & Otto 2001). Moreover, Charlesworth and Charlesworth (1978a, 1978b) have suggested that dioecious species are good competitors owing to the increased genetic variability that results from outcrossing. Dioecy is also found to be correlated with longevity (the proxy being woody growth; Renner & Ricklefs, 1995; Jaimes & Ramírez, 1999; Vamosi, Otto, & Barrett, 2003), which can increase the probability of multiple mating over time, and therefore increasing the opportunity of successful reproduction (Jaimes & Ramírez, 1999). At the metapopulation level, it has been found that population turnover was higher in hermaphrodite compared to dimorphic populations (Dorken, Freckleton, & Pannell, 2017). Thus, based on these observations, we expect dioecious species would on average would have lower rates of growth and mortality than hermaphrodite and monoecy in late successional communities.

Demographic variation within communities have been linked to a number of functional traits (Poorter et al., 2008; Wright et al., 2010; Lida et al. 2014) and ecological processes (e.g., seed dispersal and density‐dependence, Ma et al. 2016). In terms of understanding the multiple traits driving successional differences, seed mass, leaf mass per area, woody density, and maximum height of trees received a great deal of attention (Westoby et al. 2002, Westoby & Wright 2006; Poorter et al., 2008; Chave et al. 2009; Norden, Letcher, Boukili, Swenson, & Chazdon, 2012; Buzzard, Hulshof, Birt, Violle, & Enquist, 2016). However, comparative analyses of demographic performance of species with contrasting sexual systems in diverse forests at different successional stages are lacking. Further, compared with later successional stages, forest communities in early succession are usually dominated by faster‐growing species and have higher mortality and recruitment, and strongly limited by *N* and water available (Milton, Laca, & Demment, 1994; Pretzsch, 2009). However, many successional effects cannot be detected if the studies are confined solely to stands of older or younger forest (Milton et al., 1994).

Considerable variation in the response of mating system traits to the environment and habitat variation has been observed (Holtsford & Ellstrand 1992; Litrico et al., 2005; Levin, 2010; Cheptou 2012). Comparative analyses, based on correlations between breeding systems and ecological conditions, are valuable complements to studies on adaptive behavior (Leisler, Winkler, & Wink, 2002), because similar breeding system changes have evolved repeatedly (Charlesworth, 2006). Moreover, breeding systems are highly labile in angiosperm trees and often showed strong phylogenetic conservatism (Queenborough et al., 2009) as smost species evolved from outcrossing to predominant self‐fertilization (Barrett & Crowson, 2016). Monoecy in the plant lineage correlates with the evolution of dioecy (occurring in the same tree families and/or in closely related families; Renner & Ricklefs, 1995). However, phylogenetic relationships between species may influence comparisons among species in terms of responses to both biotic and abiotic conditions in natural habitats (Norden et al., 2012; Russo et al., 2010). Quantifying the relationships between demographic dynamics and breeding systems considering the effects of phylogeny at different successional stages could provide new insights into the complex interacting processes that shape community dynamics across large temporal scales (Chang & HilleRisLambers, 2016).

In this study, we use data from two long‐term temperate forest dynamics plots in Northeast China to explore the growth‐mortality dynamics in species with contrasting breeding systems at different stages of succession. We predict that (a) hermaphrodite and monoecy, on average, are disadvantageous compared to dioecy in younger successional stages with stressful conditions; in contrast; (b) in stable primary forest, dioecy should show demographic advantage over monomorphic species; (c) we predict that breeding system is linked with the trade‐off between growth and mortality; (d) dioecy should have demographic advantage in secondary forest than the primary stage. Here we present, to our knowledge, the first study to combine community assembly with community succession from both ecological and evolutionary perspectives in natural forests.

## 2 METHODS AND MATERIALS

### 2.1 Study site

The study was conducted at the Changbai Mountain Natural Reserve (42°23′N, 128°05′E) in the northeastern PR China. Two forest tree communities in different successional stages were chosen for this study. The first is a 5‐ha (250 × 200 m) secondary poplar‐birch forest naturally regenerating after clear‐cutting or fire (Bai et al. 2014). The poplar‐birch forest (~80 years) is an important stage in the secondary succession of the broad‐leaved Korean pine forest (Xu, 2001; hereafter secondary forest). The other is a 25‐ha (500 × 500 m) broad‐leaved Korean pine (*Pinus koraiensis*) mixed forest (abbreviated here as primary forest), which is the most common vegetation type in northeastern China and well known for high species richness (Stone, 2006; Yang & Xu, 2003). The climate of the study region is characterized by low temperature and high precipitation (Yang, Li, Wang, & Han, 1985). Mean annual precipitation is approximately 700 mm and most of this occurs from June to September (480–500 mm). Mean annual temperature is 2.88°C, with a January mean of 13.78°C, and a July mean of 19.63°C (Yang & Li, 1985).

### 2.2 Regular plot surveys

Both large forest dynamics plots were fully censused using the methods described by Condit (1998): all free‐standing trees ≥1 cm diameter at breast height (dbh = 1.3 m above the ground) were mapped, tagged and identified to species when the plot was established in 2005 (secondary plot) and 2004 (primary plot). The re‐censuses were carried out for secondary forest between July and August 2010 and 2015, and in 2009 and 2014 for primary forest, respectively. The status of trees (live or dead) was recorded in the re‐censuses for both plots (Zhang, 2016). More detailed information including mean stand density and mean basal area of living stems in first survey is summarized in Table 1.

**Table 1:** Summary of forest plots included in this study

Stand type (age/year)	Tree density (per hectare) and proportion (%)			Density (stems/ha)	Basal area (m^2^/ha)	Dominant species
	Dioecy	Hermaphrodite	Monoecy			
Secondary forest (~80)	318 (33.11)	1,128 (37.54)	1,159 (51.88)	4,021	28.79	*Betula platyphylla, Populus davidiana, Pinus koraiensis, Tilia amurensis, Quercus mongolica*
Primary forest (~300)	199 (14.25)	305 (21.87)	892 (63.87)	2,366	43.75	*Pinus koraiensis, Tilia amurensis, Quercus mongolica, Fraxinus mandshurica*

Note

The proportions of the species, density and basal area representing different breeding systems were the value in first census (2005 for secondary forest and 2004 for primary forest). Primary forest is the Broad‐leaved Korean pine mixed forest and secondary forest is the Poplar‐birch forest. Density = Mean stand density of living stems, basal area = Mean basal area of living stems.

### 2.3 Environmental factors

To measure water content, we first divided both study sites into a grid of number of 20 × 20 m quadrats (20 for secondary forest and 100 for primary forest, respectively). The volumetric soil water content (%) was obtained by taking three samples from each of the four corners in each quadrat across both forest plots, using a Time Domain Reflectometers (TDR) probe at a depth 20 cm. Then we took the average of six values within each 50 × 50 m. For total nitrogen, we sampled soils according to the methods of John et al. (2007): using a regular grid of points every 30 m and also selected another two sample points (2, 5 or 15 m) in a several directions (N, NE, E, SE, etc.) from each base point (Supporting Information Figure S1; John et al., 2007; Yavitt et al., 2009). At each sample location, we obtained three subsamples of 0.2 m around the point using a 5‐cm diameter cylinder at a depth of 10 cm (Yuan et al., 2013). Then we calculated the mean total nitrogen within each 50 × 50 m subplot.

### 2.4 Breeding system

The breeding system of each species was identified using the following sources in a descending order of use: *Flora of China* (http://www.eflora.cn), Seeds of *Woody Plants in China*,* Flora of Liaoning*, and a series of recent publications including Gleiser & Verdú (2005), Sun (2005), Li, Liu, and Su (2008), Zhang et al. (2010), Zhang and Shang (2010), Wang (2014), Zhu et al. (2015). For many species, it was possible to verify breeding system using multiple sources. Conflicting reports were resolved with reference to the most recent treatment.

### 2.5 Statistical analysis

We calculated demographic rates (both growth and mortality rates) from the 5‐ and 10‐year period census intervals (Sheil et al. 1995):

$$\text{RGR}\;\left(\%\right)=\frac{D_{t}-D_{0}}{D_{0}\times \Delta t}\times 100.$$

where *RGR* represents the relative growth rate, *D*_0_ and *D*_t_ are the stem diameter at breast height (dbh) from first and last measurements, respectively, *t* is the time interval:

$$m=1-{\left(\frac{N_{t}}{N_{0}}\right)}^{\frac{1}{t}}.$$

where *m* is the mean mortality per year, *N*_0_ is the stem abundance in the first census, *N*_*t*_ is the number of living stems in the *t*‐year re‐census.

Both secondary and primary forests were divided into subplots of 50 × 50 m. Thus, there were 20 and 100 subplots for secondary and primary forests, respectively. We performed species‐level analyses of tree growth and mortality by separately analyzing each of the species in all three censuses for both plots and all subplots.

### 2.6 Model construction

Breeding system has previously been shown to demonstrate significant phylogenetic conservation (Wang et al. unpublished data). The *Phylomatic* software was employed to generate the phylogeny with branch lengths provided by one of the available mega trees (http://phylodiversity.net/phylomatic/, Zanne et al. 2014). Phylogenetic analyses were conducted in the R package “*ape”* (Paradis 2004, R Development Core Team 2015).

First, to examine how the relationship between breeding system and demographic rate (growth and mortality) changes with forest successional stages [SS], we treated relative growth and mortality rates as the dependent variables separately and included breeding system as the explaining variable in the phylogenetically corrected linear mixed‐effects kinship model fit by maximum likelihood (lmekin) within the “coxme” package. To consider the environmental effects of different successional stages on demographic rate of plant individuals, we included environmental factors (water content and total Nitrogen). We also included the interaction between forest type and breeding systems of plant species, because proportions of species with different breeding systems vary across the successional stages. RGR was included as a covariate in the models of mortality rate ~ breeding system and species name was taken as a random factor.

In addition, to evaluate the role of breeding system in the trade‐off between RGR and mortality rate, and because of the well‐known effects of seed mass [SM], woody density, maximum height of trees, and leaf mass per area on growth‐mortality trade‐off (McMahon, Metcalf, & Woodall, 2011; Poorter et al., 2008; Wright et al., 2010, 2010), these covariates were also included in the models. The values of the continuous explanatory variables were standardized by subtracting the mean value of the variable and dividing by 1 standard deviation (Schielzeth 2010). Pairwise correlations showed that there was no multicollinearity following the threshold of 0.7 (Dormann et al. 2013, Supporting Information Table S1).

To test for differences in tree growth and mortality among the species with different breeding systems in each forest stage, we conducted phylogenetically generalized least squares regression (“*pgls”*) using the “*caper”* package in R (Orme, 2012). The influence of phylogenetic nonindependence of breeding systems on growth and mortality rates was modeled by incorporating the phylogenetic covariance matrix in the *pgls*. All statistics were calculated in R 3.2.2 (R Core Team 2015).

## 3 RESULTS

**Figure 1:**
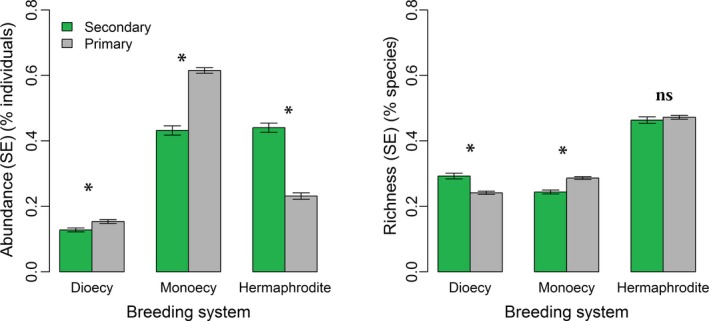
The proportions of individuals and richness (*SE*) for dioecy, hermaphrodite, and monoecy in two forest successional stages. For each breeding system, significant differences between successional stages with respect to their proportions of individuals and richness are indicated by “*” (*p *<* *0.05)

**Figure 2:**
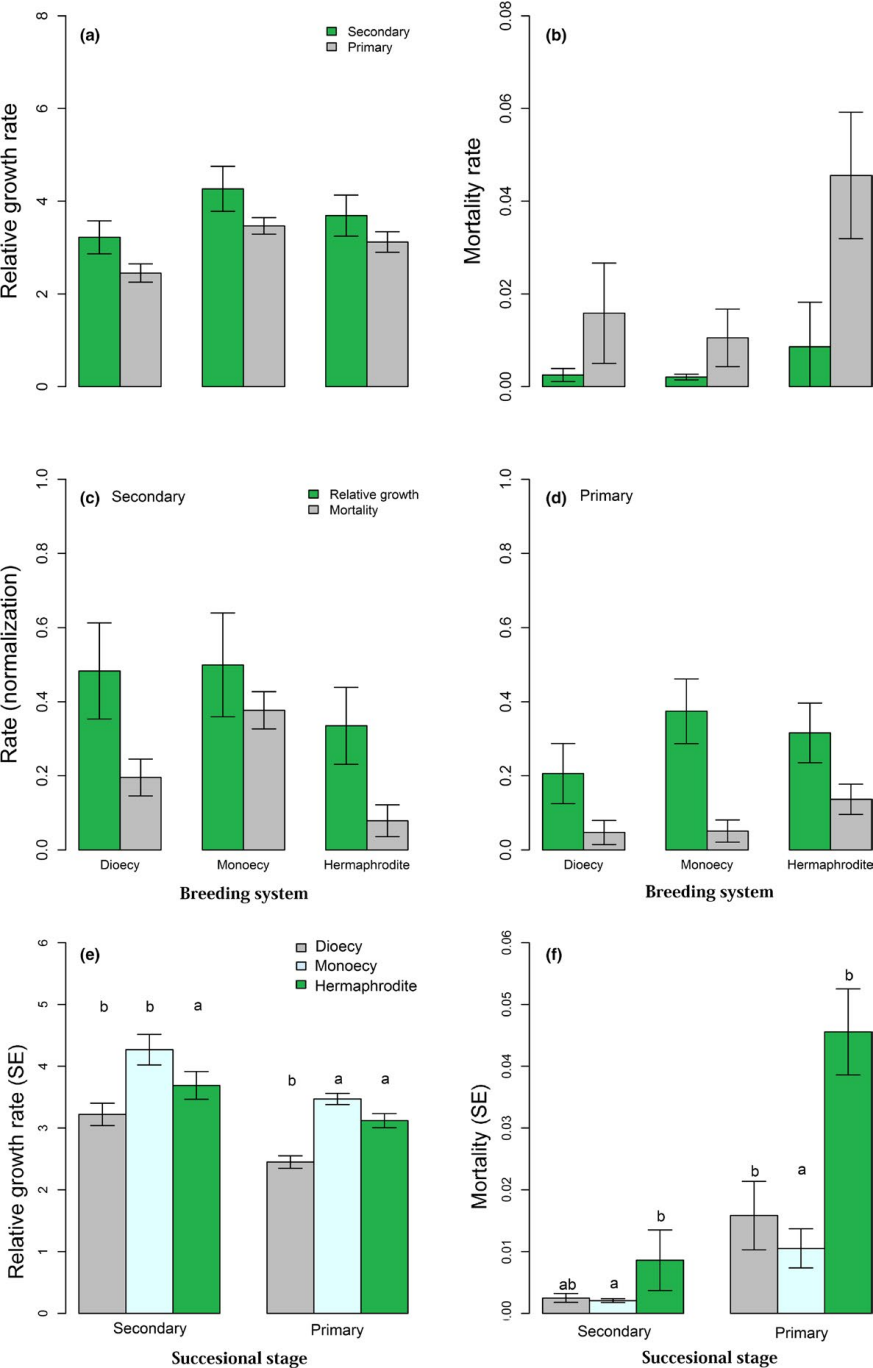
The relative growth and mortality rates between secondary and primary forests (a) and (b). (c) and (d) showed the difference between relative growth and mortality rates (normalization) at two different successional stages. (e) and (f) meant the demographic rates (relative growth rate and mortality) of species in the two forest successional stages by breeding system in *pgls* models. Within each successional stage, significant differences between breeding systems with respect to their relative growth rate and mortality rate are indicated by different letters (*p *<* *0.05)

Overall, there was significant difference in both the frequencies of abundance and species with each breeding system between two forest stages (Figure 1 and Supporting Information Figures S2 and S3). Secondary forest showed higher RGR (1.76%) and mortality rate (2.34%) than the primary forest (0.86% and 2.12%, respectively) during the 10‐year period. In secondary forest stage, RGR was negatively associated with mortality (coefficient correlation *r *=* *−0.30, *p *<* *0.01) when combining the two monitoring periods, while this relationship was significantly positive in the primary forest (*r *=* *0.33, *p *<* *0.001; Figure 2 and Supporting Information Figure S4).

Having controlled for water content, total nitrogen, seed mass, wood density, maximum height of trees, and leaf mass per area, the fitted models revealed that, on the whole, dioecious species had significantly lower RGR and mortality rates than monoecy and hermaphrodites across two forest successional stages (Table 2). The effects of being monoecious or hermaphrodite on RGR and mortality rate were much stronger than the leaf mass per area, woody density and maximum height. The interaction between hermaphrodite and secondary stage of forest had a stronger effect on both RGR and mortality rate (Table 2).

**Table 2:** Summary of phylogenetically corrected linear models designed to detect effects of breeding systems on relative growth rate and mortality rate for both forest types during 10 years. Values in bold are significant at *P* < 0.05

Parameter	Relative growth rate			Mortality rate		
	Value	*SE*	*p*‐value	Value	*SE*	*p*‐value
Hermaphrodite	1.887	0.350	**<0.0001**	0.005	0.001	**<0.0001**
Monoecy	2.258	0.520	**<0.0001**	0.003	0.002	**0.022**
Water contain	0.033	0.072	0.640	0.001	<0.001	**0.034**
Total nitrogen	0.165	0.065	**0.011**	<0.001	<0.001	0.330
Leaf mass per area	−0.605	0.202	**0.003**	0.001	0.001	0.240
Woody density	−0.919	0.164	**<0.0001**	−0.002	0.001	**<0.0001**
Maximum height	−0.363	0.279	0.190	−0.003	0.001	**<0.001**
Seed mass	−0.087	0.322	0.790	0.002	0.001	0.053
Secondary	1.227	0.238	**<0.0001**	−0.001	0.001	0.370
Hermaphrodite : secondary	−0.869	0.294	**0.003**	−0.003	0.001	**0.019**
Monoecy: secondary	−0.148	0.322	0.650	−0.003	0.002	**0.042**
Relative growth rate				−0.0004	<0.001	0.190

Note

The models controlled for water content, total nitrogen, seed mass, wood density, maximum height of trees, leaf mass per area, successional stage, and the interactions between breeding system and successional stages.

In secondary forest, RGR differed little among dioecious species, monoecious species, and hermaphrodite during 10 years when controlling phylogeny, while hermaphroditic species had (marginally not) significantly higher mortality rate (Tables 3 and 4). By contrast, phylogenetic independence analysis showed that dioecy had higher relative growth rate and lower mortality rate compared to hermaphrodite (Supporting Information Figure S4). In primary forest, hermaphroditic species had both significantly higher RGR and mortality rates than both dioecious and monoecious ones (*p *<* *0.05, Figure 2e,f; Tables 3 and 4). Phylogenetic independence analysis revealed similar results (Supporting Information Figure S4). Further comparison showed that the difference between RGR and mortality rate for dioecious species between two successional stages was insignificantly (Figure 2c,d).

**Table 3:** Summary of Phylogenetic Generalized Least Square models designed to detect effects of breeding systems on relative growth rates for both forests during 10 years. Values in bold are significant at *P* < 0.05

Study period	Parameter	Secondary forest				Primary forest			
		Estimate	*SE*	*p*_e_	*p*_m_	Estimate	*SE*	*p*_e_	*p*_m_
Period 1	Hermaphrodite	−0.447	0.827	0.590	0.762	1.944	0.493	**<0.0001**	0.007
	Monoecy	1.205	1.078	0.260		0.490	0.747	0.510	
Period 2	Hermaphrodite	−0.365	0.505	0.470	0.127	1.193	0.318	**<0.001**	<0.001
	Monoecy	−0.419	0.885	0.640		0.727	0.471	0.120	
10 years	Hermaphrodite	1.472	0.655	**0.025**	0.545	2.339	0.788	**0.003**	<0.001
	Monoecy	0.958	0.900	0.290		1.956	1.501	0.190	

Note

*p* Showed the significance of the difference in relative growth rate between breeding systems. And Estimates showed the difference between dioecious and nondioecious species. Period 1 indicates 2005–2010 for secondary forest and 2004–2009 for primary forest; period 2 indicates 2010–2015 for secondary forest and 2009–2014 for primary forest. In accordance with, the 10 years are 2005–2015 for secondary forest and 2004–2014 for primary forest.

**Table 4:** Summary of Phylogenetic Generalized Least Square models designed to detect the difference in terms of the effects of breeding systems on mortality rates for both forests during 10 years. Values in bold are significant at *P* < 0.05

Study period	Parameter	Secondary forest				Primary forest			
		Estimate	*SE*	*p*_e_	*p*_m_	Estimate	*SE*	*p*_e_	*p*_m_
Period 1	Hermaphrodite	−0.033	0.043	0.450	<0.0001	0.051	0.018	**0.005**	<0.001
	Monoecy	0.027	0.042	0.520		−0.004	0.025	0.880	
Period 2	Hermaphrodite	0.068	0.042	0.100	<0.001	0.062	0.027	**0.024**	<0.01
	Monoecy	−0.005	0.052	0.920		0.014	0.040	0.720	
10 years	Hermaphrodite	0.006	0.005	0.190	<0.001	0.070	0.028	**0.011**	<0.01
	Monoecy	<0.001	0.006	0.940		−0.050	0.042	0.240	

Note

*p* Showed the significance of the difference in mortality rate between breeding systems. And Estimates showed the difference between dioecious and nondioecious species. Period 1 indicates 2005–2010 for secondary forest and 2004–2009 for primary forest; period 2 indicates 2010–2015 for secondary forest and 2009–2014 for primary forest. In accordance with, the 10 years are 2005–2015 for secondary forest and 2004–2014 for primary forest.

## 4 DISCUSSION

In this study, we evaluated growth and mortality rates in two permanent temperate forest dynamics plots at different successional stages in Northeast China and found that demographic rates were linked with variation in breeding system. Interspecific variation in RGR was negatively correlated with mortality rate in the early successional forest. In contrast, this demographic trade‐off between growth and mortality was positive in the primary forest community. We have provided weak evidence that in the younger successional stages, dioecious species have weak demographic advantage over monomorphic species. And dioecious species showed on average lower rates of growth and mortality than hermaphrodite (thus higher demographic advantage for dioecy) in the primary community. Comparison between two stages showed that dioecy also had no demographic advantage in secondary forest than the primary stage. Nevertheless, our results also revealed that breeding system, together with environmental factors, and other well‐known functional traits (e.g., seed mass and leaf mass per area), contributed to the growth‐mortality trade‐off in plant communities and played a greater role compared with those functional traits.

### 4.1 Trade‐off between growth and mortality as succession proceeds

In the secondary forest community, we found that RGR and mortality rate were negatively correlated. This is consistent with previous findings in other secondary forest with a comparable mean basal area (28 m^2^/ha) as our 5‐ha plot: as tree growth rate declines, the probability of mortality increases (Monserud, 1976; Wyckoff & Clark, 2002). In stark contrast, in primary forest, the interspecific variation in RGR was positively correlated with mortality rate, in accordance with previous studies (Poorter et al., 2008; Wright et al., 2010; Iida et al. 2014). In general, forests at early stages are in the process of accumulating biomass rapidly (Van Breugel, Martínez‐Ramos, & Bongers, 2006). As the vegetation develops, taller longer‐lived plant species in late successional stages progressively replace the species which are faster‐growing, having higher mortality, recruitment, stem density, and lower basal area (Garnier et al., 2004). Thus, both RGR and mortality rate decline with increasing tree size (Wyckoff & Clark, 2002; Iida et al. 2014) and stand age (Xu et al., 2012).

### 4.2 Demographic comparison between breeding systems in earlier forest

Inconsistent with this prediction, there was only weak demographic advantage in terms of both RGR and mortality rates for dioecious compared with hermaphrodite species in the secondary forest community where dioecious species (richness here) are more common than in the old‐growth. That is, in relatively newly colonized areas which are relatively stressful environments, monomorphic species showed no demographic advantage over dioecious species, although a single self‐compatible immigrant can initiate a sexually reproducing colony without the aid of an external agent for pollination (Baker, 1955, 1967). Harsh or stressful conditions have been considered to favor the evolution and maintenance of separate sexes from combined sexes (Barrett, Dorken & Case 2001; Ashman, 2006). Therefore owing to the limited resources availability for plant growth, there is a trade‐off between reproduction and growth (Harper, 1977; Obeso, 2002), especially in stressful environments (Vilà‐Cabrera, Martínez‐Vilalta, & Retana, 2014). This may be consistent with our findings that dioecy had a higher proportion of richness but lower proportion of abundance in secondary forest. This suggests that the demographic advantage of monomorphic species over dioecious species is reduced in disturbed areas, most likely by a lower investment in the female function of hermaphroditic species (Costich, 1995; Litrico et al., 2005). This is because female function is much more costly than male function (Freeman, Doust, El‐Keblawi, Miglia, & McArthur, 1997; Shelton, 2010). The adaptive strategies of dioecy in disturbed areas contributed to a demographic advantage for dioecy and a comparable species richness for both breeding systems in early successional stages.

### 4.3 Demographic comparison between breeding systems in primary forest

Dioecious species showed lower rates of growth and mortality than hermaphrodite and monoecious ones in the primary forest community, supporting prior predictions. These results suggest that dioecious species had a demographic advantage over the cosexual ones, which may reflect that the dioecious species were better competitors in terms of survival than the hermaphrodite to some degree (Kunstler et al., 2016). It might be expected that being unisexual would reduce inbreeding depression and prevent competition for resources between male and female flowers (Charlesworth & Charlesworth, 1978a, 1978b), thus obligate outcrossing of dioecious species has been viewed as the trade‐off for the loss of effective reproductive population (Charlesworth & Charlesworth, 1978a, 1978b; Lloyd, 1979). In contrast, most cosexual individuals, experiencing inbreeding depression, are less competitive at every stage of their life history (Charlesworth & Charlesworth, 1978a, 1978b; Sakai, Karoly, & Weller, 1989). This competitive advantage may possibly contribute to the coexistence of dioecious plants and other breeding systems in the absence of fecundity and population density advantages in primary forest communities (Vamosi, Mazer, & Cornejo, 2008; Queenborough et al., 2009; Yunyun Wang et al. unpublished).

### 4.4 The role of breeding system in the trade‐off between growth and mortality

The trade‐off between growth and mortality has been found to be associated with a number of functional traits that relate to the ability to disperse (seed mass), acquire resources (leaf mass per area), and grow rapidly (woody density; Liu et al., 2016; McMahon et al., 2011; Poorter et al., 2008; Wright et al., 2010). The analysis presented here revealed that sexual system (e.g., dioecy and hermaphrodite) was one of the key traits correlated with RGR and mortality rate across successional stages of forest (Table 2, Figures 2, Supporting Information Figure S5), which suggests that breeding system played a significant role in driving demographic rates and community dynamics. The growth‐mortality relationship represents a trade‐off between a species’ ability to grow quickly to exploit abundant resources versus avoiding mortality when resources are scarce (Russo, Brown, Tan, & Davies, 2008). In early successional areas with stressful conditions, hermaphrodites would decrease the emphasis on seed production to avoid more waste of resources (Ashman, 2006). In the primary forest in which various species begin to compete for space, light, and nutrients (Clements 1916), while adaptations for fast growth can provide a competitive advantage by way of rapid exploitation of resources for hermaphrodite, an inability to acclimate to reduced resource availability could increase mortality risk (Russo et al., 2008).

### 4.5 Stage‐dependence of breeding systems in demographic dynamics

It is interesting that our results indicated that there is a switching of difference in demographic rates between dioecious and hermaphroditic species as succession proceeds. This phenomenon is likely to reflect the response of the sexual system to the contrasts in stem density, canopy structure characteristics, and biomass level as the succession proceeds and becomes well established. Forests at secondary stage are likely at higher tree density (greater frequency of colonists/pioneers) of small diameter and face intense competition for abundant resources (e.g., light; Milton et al., 1994), while the importance of competition increases with succession because resources become limited as stand biomass increases (Grubb, 1977). Such increased competitive pressure will lead to differentiated strategies to obtain the increasingly scarce resources, with concomitant different functional traits, a progress leading to increasingly limited trait similarity (Lohbeck et al., 2014). Such trait differentiation over habitat gradients may also apply to sexual systems: changing sex ratio (equal sex ratios or male‐biased) could benefit dioecious species (Queenborough, Burslem, Garwood, and Valencia (2007). In some species, females show different or stronger habitat associations than males. This differences in physiology and function between female and male in certain setting may partly contribute to the transformation from a similar demographic rate between dioecy and hermaphrodite in secondary forest to a significant difference in primary forest.

Ecological variables that are associated with resources available for pollinators (e.g., quantity of inflorescence) have a great impact on population structure through their effect on the pollinator behavior and plant mating (Case & Ashman, 2009; Gervasi & Schiestl, 2017; Iwata, Nagasaki, Ishii, & Ushimaru,^2012^). In addition, the sex ratio of species can vary considerably in response to tree size, density and stand age if the plants are growing in a stressful environment (Charlesworth, 2006; Lloyd & Bawa, 1984; Ortiz, Arista, & Talavera, 2002), which likely explains the frequency and role changing of hermaphrodite over succession. A study analyzing the sex ratios of 126 dioecious species has showed nearly continuous variation from strong male to strong female bias, and exhibit male‐biased sex ratios in some younger populations, but close to even sex ratios in older populations in which all individuals were reproductively primary (Barrett, Yakimowski, Field, & Pickup, 2010). In an ultimate manner, the changing sex ratio was attributed to the longer time required for females to reach reproductive maturity because of costs associated with serotinous cone production (Harris & Pannell, 2010), in which significant resource is also invested.

### 4.6 Caveats

Forest succession is a long process that includes a series of stages as its disturbance. Within or between different successional stages, one or several particular forest successional stage(s) could reflect some growth and mortality dynamics in breeding systems. In general, community‐assembly studies have gradually considered sexual systems, together with the development of long‐term forest dynamics plots across the world (http://www.ctfs.si.edu/), ongoing efforts will constitute a very strong test of patterns arising during succession.

## 5 CONCLUSION

Breeding system has been considered to play an important role in community dynamics and assembly, but how it contributes and changes community dynamics during succession has not been tested. Here, we have shown that plant breeding system is strongly linked with demographic rates in two temperate forest communities at different successional stages. Demographic rates are clearly stand‐dependent (Harper, 1977). It is interesting that there is then a switch in ecological advantage of dioecious species in different forests: a marginally similar demographic rate between dioecy and hermaphrodite in secondary forest to a significant difference in primary forest.

We propose that breeding systems that relate to colonization propensity and competitive ability are closely associated with the growth‐mortality trade‐off in both early and late successional forests, which may be a key mechanism driving the community succession and assembly (Réjou‐Méchain & Cheptou, 2015). These results improve our understanding of how breeding system links to demographic performance, and community successional, dynamics, and assembly.

## AUTHOR CONTRIBUTIONS

Wang, Y.Y. and Freckleton, R.P. conceived the ideas and designed methodology. Wang, Y.Y., Wang, B.J., Kuang, X., Yuan, Z.Q., Lin, F., Ye, J., Wang, X.G. and Hao, Z.Q. performed the experiments. Wang, Y.Y. and Kuang, X. analyzed the data, Freckleton, R.P. consulted on data analyses. Wang, Y.Y. led the writing of the manuscript, Freckleton, R.P. contributed critically to the drafts. All authors gave final approval for publication.
