# Supplementary material for: The role of breeding system in community dynamics: Growth and mortality in forests of different successional stages
Source: Ecol Evol. 2018 Jun 27;8(15):7285–96. doi: 10.1002/ece3.4190 (PMC6106203; doi:10.1002/ece3.4190)

**Supporting information**

**Table S1** Pairwise relationships among demographic rates (relative growth rate-RGR and mortality rate) and functional traits including maximum height (*H*_max_), wood density (WD), Seed Mass, and leaf mass per area (LMA) for trees in secondary and primary forests in temperate Changbai Mountain, Northeast China.

| Secondary forest | Variables | RGR | Mortality | SeedMass | WD | *H*_max_ | LMA |
| --- | --- | --- | --- | --- | --- | --- | --- |
|  | RGR |  |  | -0.066 | -0.148 | -0.101 | -0.184 |
|  | Mortality | -0.285^**^ |  | -0.037 | -0.058 | -0.354* | 0.121 |
|  | SeedMass | -0.066 | -0.037 |  | 0.245* | 0.19 | 0.135 |
|  | WD | -0.148 | -0.058 | 0.245^*^ |  | -0.257* | -0.242 * |
|  | *H*_max_ | -0.101 | -0.354^***^ | 0.191 | -0.257^*^ |  | 0.626*** |
|  | LMA | -0.184 | 0.121 | 0.135 | -0.242^*^ | 0.626^***^ |  |
| Primary forest | Variables | RGR | Mortality | SeedMass | WD | *H*_max_ | LMA |
|  | RGR |  | 0.229* | -0.143 | 0.024 | -0.463*** | -0.280** |
|  | Mortality | 0.229* |  | -0.091 | 0.141 | -0.499*** | -0.08 5 |
|  | SeedMass | -0.143 | -0.091 |  | 0.193* | 0.133 | 0.107 |
|  | WD | 0.024 | 0.141 | 0.193* |  | -0.387*** | -0.416*** |
|  | *H*_max_ | -0.463*** | -0.499*** | 0.133 | -0.387*** |  | 0.662*** |
|  | LMA | -0.280** | -0.085 | 0.107 | -0.416*** | 0.662*** |  |

**Table S2** The list of species measured in the study. We classified all these species into one of the three breeding systems: dioecy (separate male and female individuals), monoecy, and hermaphrodite (spatial separation of investment in male and female function) based on the classification by Gross et al. (2005). The dioecy includes all the androdioecious species, while the monoecy includes the andromonoecious species.

| Genus_species | Breeding System | Original Breeding System |
| --- | --- | --- |
| *Betula platyphylla* | Monoecy | Monoecy |
| *Acer mandshuricum* | Dioecy | Androdioecy |
| *Syringa reticulata* | Hermaphrodite | Hermaphrodite |
| *Lonicera tatarinowii* | Hermaphrodite | Hermaphrodite |
| *Acer ginnala* | [Monoecy](http://www.dictall.com/indu/170/16991419840.htm) | [Andromonoecy](http://www.dictall.com/indu/170/16991419840.htm) |
| *Euonymus macropterus* | Hermaphrodite | Hermaphrodite |
| *Prunus racemosa* | Hermaphrodite | Hermaphrodite |
| *Ulmus davidiana var. japonica* | Hermaphrodite | Hermaphrodite |
| *Acanthopanax senticosus* | Hermaphrodite | Hermaphrodite |
| *Acer barbinerve* | Dioecy | Dioecy |
| *Populus ussuriensis* | Dioecy | Dioecy |
| *Lonicera subhispida* | Hermaphrodite | Hermaphrodite |
| *Prinsepia sinensis* | Hermaphrodite | Hermaphrodite |
| *Ribes mandshuricum* | Hermaphrodite | Hermaphrodite |
| *Philadelphus schrenkii* | Hermaphrodite | Hermaphrodite |
| *Deutzia parviflora var. amurensis* | Hermaphrodite | Hermaphrodite |
| *Acanthopanax sessiliflorus* | Hermaphrodite | Hermaphrodite |
| *Betula costata* | Monoecy | Monoecy |
| *Actinidia kolomikta* | Dioecy | Dioecy |
| *Salix matsudana* | Dioecy | Dioecy |
| *Juglans mandshurica* | Monoecy | Monoecy |
| *Sorbus Pohuashanensis* | Hermaphrodite | Hermaphrodite |
| *Maackia amurensis* | Hermaphrodite | Hermaphrodite |
| *Phellodendron amurense* | Dioecy | Dioecy |
| *Lonicera chrysantha* | Hermaphrodite | Hermaphrodite |
| *Viburnum opulus* | Hermaphrodite | Hermaphrodite |
| *Acer pseudosieboldianum* | [Monoecy](http://www.dictall.com/indu/170/16991419840.htm) | [Andromonoecy](http://www.dictall.com/indu/170/16991419840.htm) |
| *Ribes maximowiczianum* | Dioecy | Dioecy |
| *Sambucus williamsii* | Hermaphrodite | Hermaphrodite |
| *Rhamnus diamantiaca* | Dioecy | Dioecy |
| *Tilia mandshurica* | Hermaphrodite | Hermaphrodite |
| *Ulmus laciniata* | Hermaphrodite | Hermaphrodite |
| *Euonymus verrucosus* | Hermaphrodite | Hermaphrodite |
| *Aralia elata* | Hermaphrodite | Hermaphrodite |
| *Crataegus maximowiczii* | Hermaphrodite | Hermaphrodite |
| *Corylus mandshurica* | Monoecy | Monoecy |
| *Quercus mongolica* | Monoecy | Monoecy |
| *Acer triflorum* | Dioecy | Androdioecy |
| *Viburnum burejaeticum* | Hermaphrodite | Hermaphrodite |
| *Acer tegmentosum* | [Monoecy](http://www.dictall.com/indu/170/16991419840.htm) | [Andromonoecy](http://www.dictall.com/indu/170/16991419840.htm) |
| *Lonicera japonica* | Hermaphrodite | Hermaphrodite |
| *Actinidia arguta* | Dioecy | Dioecy |
| *Acer mono* | [Monoecy](http://www.dictall.com/indu/170/16991419840.htm) | [Andromonoecy](http://www.dictall.com/indu/170/16991419840.htm) |
| *Rosa davurica* | Hermaphrodite | Hermaphrodite |
| *Malus baccata* | Hermaphrodite | Hermaphrodite |
| *Pyrus ussuriensis* | Hermaphrodite | Hermaphrodite |
| *Vitis amurensis* | Dioecy | Dioecy |
| *Populus davidiana* | Dioecy | Dioecy |
| *Cerasus maximowiczii* | Hermaphrodite | Hermaphrodite |
| *Rhamnus davurica* | Dioecy | Dioecy |
| *Fraxinus mandschurica* | Dioecy | Dioecy |
| *Sorbus alnifolia* | Hermaphrodite | Hermaphrodite |
| *Euonymus alatus* | Hermaphrodite | Hermaphrodite |
| *Rhamnus ussuriensis* | Dioecy | Dioecy |
| *Schisandra chinensis* | Dioecy | Dioecy |
| *Populus koreana* | Dioecy | Dioecy |
| *Acer komarovii* | Dioecy | Dioecy |
| *Crataegus sanguinea* | Hermaphrodite | Hermaphrodite |
| *Salix floderusii* | Dioecy | Dioecy |
| *Lonicera ruprechtiana* | Hermaphrodite | Hermaphrodite |
| *Sorbaria sorbifolia* | Hermaphrodite | Hermaphrodite |
| *Tilia amurensis* | Hermaphrodite | Hermaphrodite |
| *Chosenia arbutifolia* | Dioecy | Dioecy |

**Figure S1**. The soil sample distribution pattern of the 25-ha (500 × 500 m) Changbai temperate forest plot.


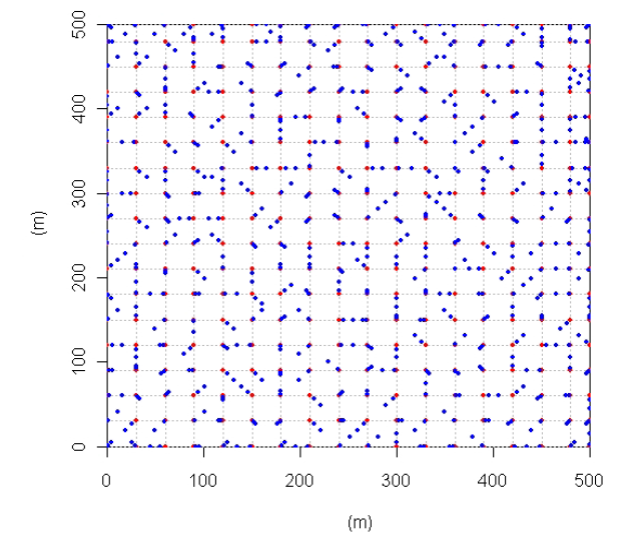


**Figure S2** Histogram of the percentage of dioecy (% of individuals and richness) within the 100 1-ha primary and 20 1-ha ﬁeld plots.


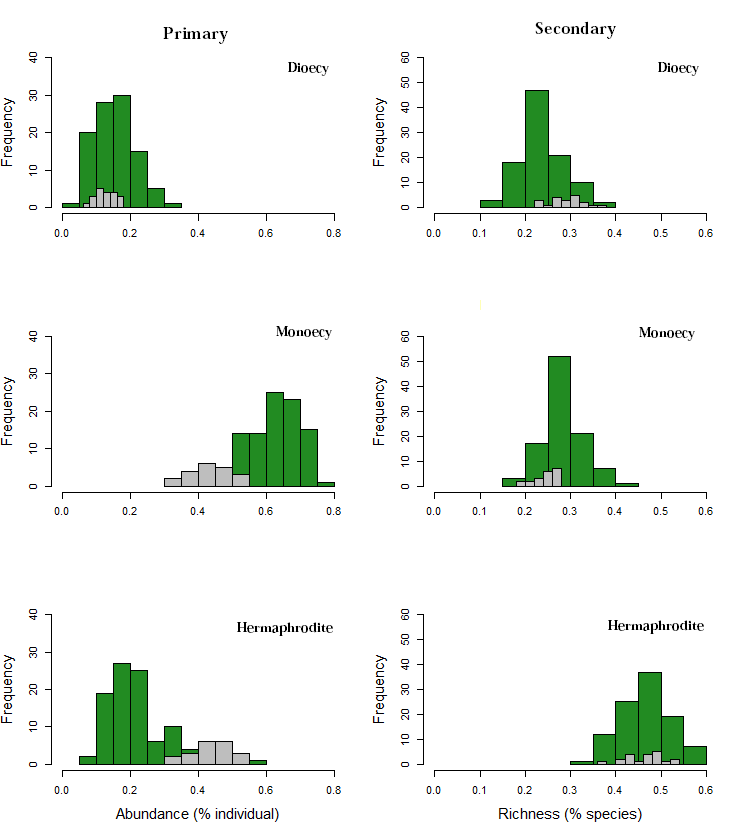


**Figure S3** Histogram of the percentage of relative growth and mortality rates within the 100 1-ha primary and 20 1-ha ﬁeld plots.


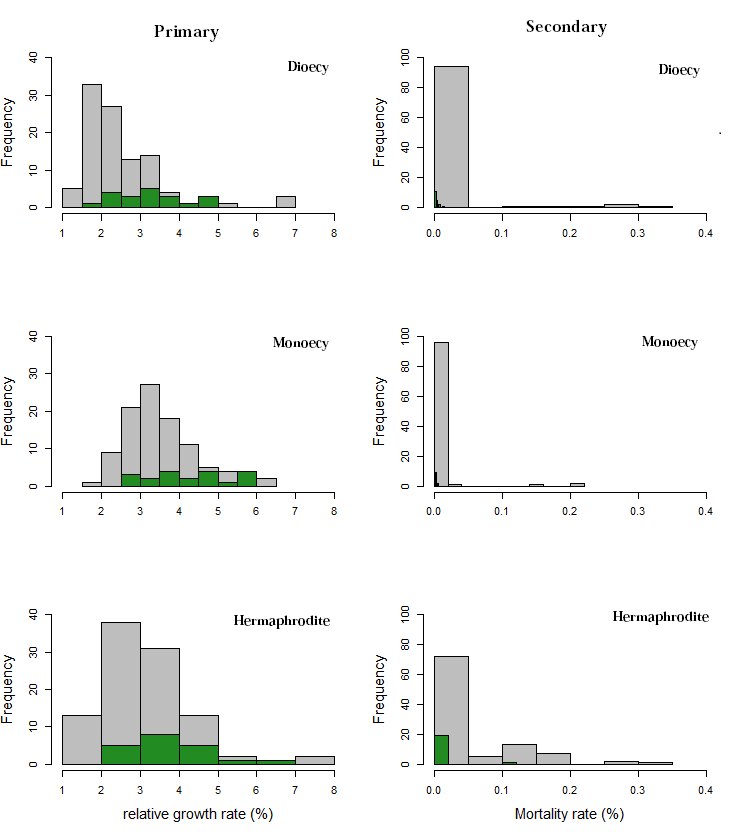


**Figure S4**. The relationships between relative growth and mortality rates in both secondary and primary forests. Both datasets during two periods (2005-2010 and 2010-2015 for secondary forests and 2004-2009 and 2009-2014 for primary forests) are combined.


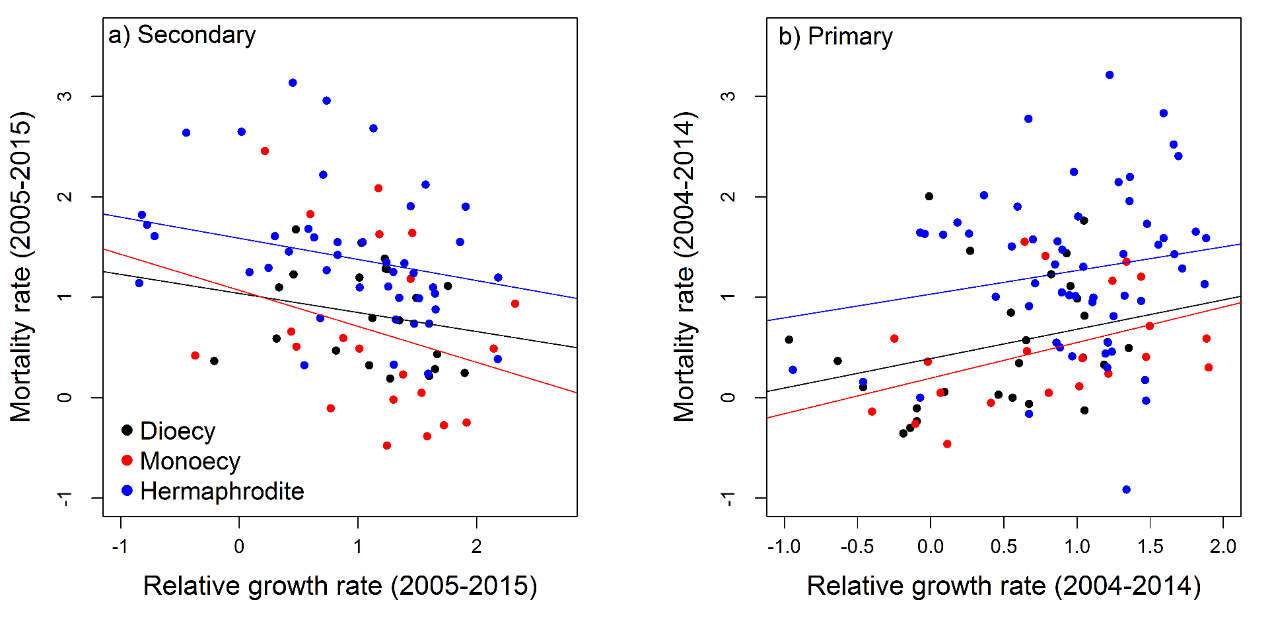


**Figure S5**. Odds ratios of a) secondary and b) primary forests for the full *pgls* models. Circles show odds ratios for each parameter, with 95 % confidence limits (CL) indicated by horizontal lines. Odds ratios significantly different from 1 (95 % CL do not overlap 1) are indicated by filled circles.


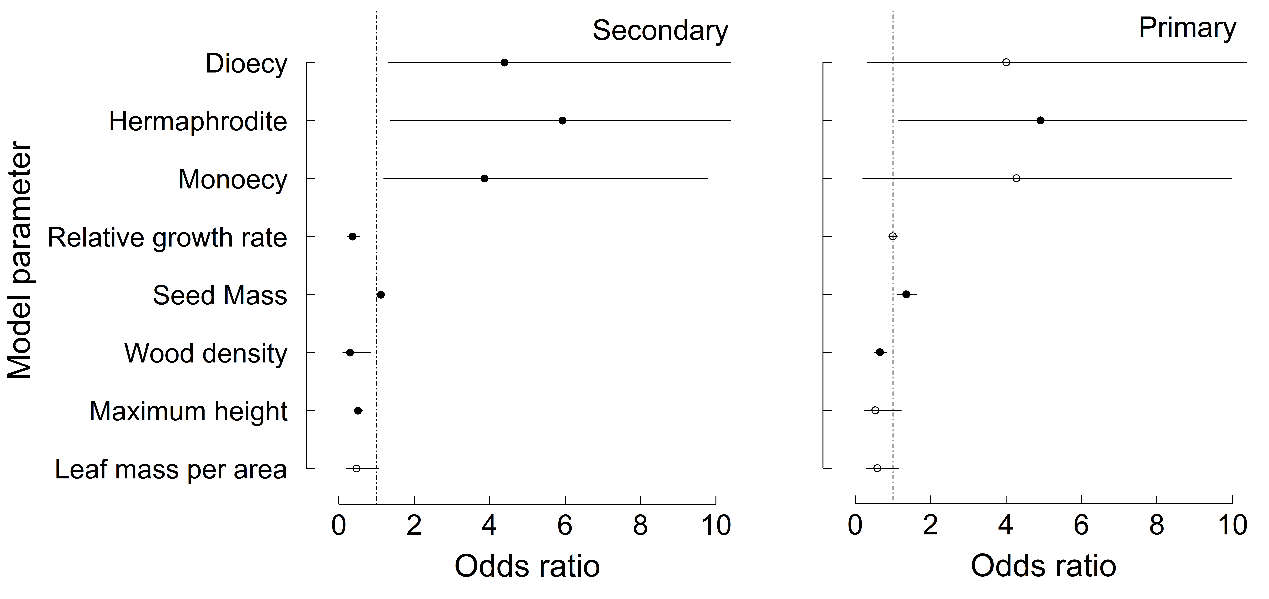

Supplement: Supplementary file 1 [file ECE3-8-7285-s001.docx]
